# *Arabidopsis FHY3* and *FAR1* Function in Age Gating of Leaf Senescence

**DOI:** 10.3389/fpls.2021.770060

**Published:** 2021-10-28

**Authors:** Yurong Xie, Mengdi Ma, Yang Liu, Baobao Wang, Hongbin Wei, Dexin Kong, Haiyang Wang

**Affiliations:** ^1^Biotechnology Research Institute, Chinese Academy of Agricultural Sciences, Beijing, China; ^2^State Key Laboratory for Conservation and Utilization of Subtropical Agro-Bioresources, South China Agricultural University, Guangzhou, China; ^3^Guangdong Laboratory for Lingnan Modern Agriculture, Guangzhou, China

**Keywords:** *Arabidopsis*, leaf senescence, *FHY3/FAR1*, EIN3, PIF5, *ORE1*

## Abstract

Leaf senescence is the terminal stage of leaf development. Both light and the plant hormone ethylene play important roles in regulating leaf senescence. However, how they coordinately regulate leaf senescence during leaf development remains largely unclear. In this study, we show that FHY3 and FAR1, two homologous proteins essential for phytochrome A-mediated light signaling, physically interact with and repress the DNA binding activity of EIN3 (a key transcription factor essential for ethylene signaling) and PIF5 (a bHLH transcription factor negatively regulating light signaling), and interfere with their DNA binding to the promoter of *ORE1*, which encodes a key NAC transcription factor promoting leaf senescence. In addition, we show that FHY3, PIF5, and EIN3 form a tri-protein complex(es) and that they coordinately regulate the progression of leaf senescence. We show that during aging or under dark conditions, accumulation of FHY3 protein decreases, thus lifting its repression on DNA binding of EIN3 and PIF5, leading to the increase of *ORE1* expression and onset of leaf senescence. Our combined results suggest that FHY3 and FAR1 act in an age gating mechanism to prevent precocious leaf senescence by integrating light and ethylene signaling with developmental aging.

## Introduction

Leaf senescence is the last stage of leaf development, during which macromolecules (such as nucleic acids and proteins) are degraded in an orderly fashion, and the resulting nutrients are mobilized from old leaves to actively growing tissues or storage organs, thus increasing plant fitness (Lim et al., 2007). Leaf senescence can be conceptually divided into three phases: initiation (onset), reorganization (progression) and termination (completion), with each process being tightly regulated by genetic, developmental and environmental factors (Gan and Amasino, 1997; Nam, 1997; Li et al., 2018).

Over the past few decades, the utilization of genetic and molecular biology approaches, and more recently, the use of multi-omics technologies together with computational biology tools have greatly aided in the identification of key players and the associated gene regulatory networks (GRNs) regulating the various processes of leaf senescence (Woo et al., 2019). Particularly illuminating, several GRNs of NAC (NAM/ATAF/CUC) and WRKY transcription factors have been shown to change dynamically as leaf senescence progresses (Kim et al., 2016). For instance, it has been shown that the GRNs involving the NAC transcription factor ORE1 (ORESARA1, means “long living” in Korean) play an essential role in promoting leaf senescence (Oh et al., 1997; Park et al., 2019). ORE1 protein promotes leaf senescence by directly activating the expression of numerous chlorophyll catabolic genes (*CCGs*), such as *NYE1* (*NON-YELLOWING 1*), *NYC1* (*NON-YELLOW COLORING 1*), and *PAO* (*PHEOPHORBIDE A OXYGENASE*), and senescence associated genes (*SAGs*) on one hand (Qiu et al., 2015), and on the other hand, inhibits the function of the chloroplast maintenance factor GLK1 (GOLDEN-LIKE 1) via protein-protein interaction (Rauf et al., 2013). Expression of *ORE1* in young leaves is repressed at the posttranscriptional level, and during aging, the repression of *ORE1* expression is alleviated due to age-dependent down-regulation of *MIR164* expression by EIN2 (ETHYLENE INSENSITIVE 2, a key regulator essential for ethylene signaling) (Kim et al., 2009). It has also been shown that expression of *ORE1* is positively regulated by several transcription factors, including EIN3/EIL1 (ETHYLENE INSENSITIVE 3/EIN3-LIKE 1), ATAF1 (ARABIDOPSIS TRANSCRIPTION ACTIVATION FACTOR 1), ABI5/EEL (ABA INSENSITIVE 5/ENHANCED EM LEVEL), and PIF4/5 (PHYTOCHROME-INTERACTING FACTOR 4/5) (Li et al., 2013; Sakuraba et al., 2014; Song et al., 2014). Thus, ORE1 acts in multiple coherent feed-forward loops to promote leaf senescence by integrating signals from ethylene, abscisic acid (ABA), salinity and light/dark into developmental aging.

Light is a key environmental factor influencing the onset and progress of leaf senescence. Darkness (light deprivation), low intensity of light or shade (low Red: Far-Red ratios) are known to induce leaf senescence (Lim et al., 2007; Brouwer et al., 2012, 2014; Liebsch and Keech, 2016). Recent studies have shown that in *Arabidopsis*, the red light photoreceptor phyB plays a role in inhibiting leaf senescence (Sakuraba et al., 2014), whereas in far red light enriched environment, phyA represses but phyB induces leaf senescence (Lim et al., 2018). In addition, recent studies showed that a group of bHLH proteins named phytochrome-interacting factors (PIFs) also promote leaf senescence. PIF4 and PIF5, whose protein accumulation is stimulated by darkness or shade, can directly activate the expression of *EIN*3 and *ORE1* to promote leaf senescence (Sakuraba et al., 2014). In another study, it was shown that PIF4 regulates chlorophyll degradation, chloroplast activity, dark-induced ethylene biosynthesis and ethylene-induced leaf senescence (Song et al., 2014). These studies suggest that light and ethylene signaling pathways converge on *EIN3* and *ORE1* to regulate leaf senescence. Furthermore, recent studies showed that leaf senescence is also regulated by the circadian clock. For example, it was shown that the evening complex (EC) can directly regulate the expression of *MYELOCYTOMATOSIS-RELATED PROTEIN 2* (*MYC2*), a key transcription factor mediating jasmonates (JA)-induced leaf senescence (Zhang et al., 2018). In another study, it was reported that PRR9 (PSEUDO-RESPONSE REGULATOR 9), a key component of the circadian clock, directly regulates the expression of *ORE1* and *MIR164*, thus forming a feed-forward loop regulating leaf senescence (Kim et al., 2018). Despite the progress made in this field, the detailed molecular mechanisms of light signaling regulating leaf senescence, particularly how light signaling integrates with ethylene signaling and developmental aging to coordinately regulate the onset of leaf senescence, still remain largely unclear.

*Arabidopsis FHY3* (*FAR-RED ELONGATED HYPOCOTYL 3*) and *FAR1* (*FAR-RED IMPAIRED RESPONSE 1*) were initially identified as two positive regulators of phytochrome A signaling and far-red light mediated photomorphogenic development (Hudson et al., 1999; Wang and Deng, 2002). They encode two homologous transcription factors derived from transposase and they regulate phyA signaling by direct activating the expression of *FHY1* (*FAR-RED ELONGATED HYPOCOTYL 1*) and *FHL* (*FHY1-LIKE*), whose gene products encodes two homologous chaperone proteins required for light-induced phyA nuclear translocation (Lin et al., 2007). Follow-up studies have demonstrated that FHY3 and FAR1 also play a wide range of biological roles, including UV-B signaling, circadian clock entrainment and flowering, chloroplast biogenesis and chlorophyll biosynthesis, ABA signaling and branching (Wang and Wang, 2015). Recently, it was reported that *FHY3* and *FAR1* also regulate leaf senescence by directly repressing the expression of *WRKY28* and salicylic acid (SA) biosynthesis (Tian et al., 2020).

In this study, we demonstrate that FHY3 and FAR1 repress leaf senescence by physically interacting with EIN3 and PIF5, and inhibiting their transcription activation activity on *ORE1* and other *SAGs*. Our results expand the functional roles of *FHY3* and *FAR1*, and deepen our understanding of the molecular mechanisms regulating leaf senescence through integration of the light and ethylene signaling pathways. Our results suggest that *FHY3* and *FAR1* act in an age gating mechanism to prevent precocious leaf senescence.

## Results

### *FHY3* and *FAR1* Repress Leaf Senescence and Depend on *EIN3* and *EIL1*

**FIGURE 1:**
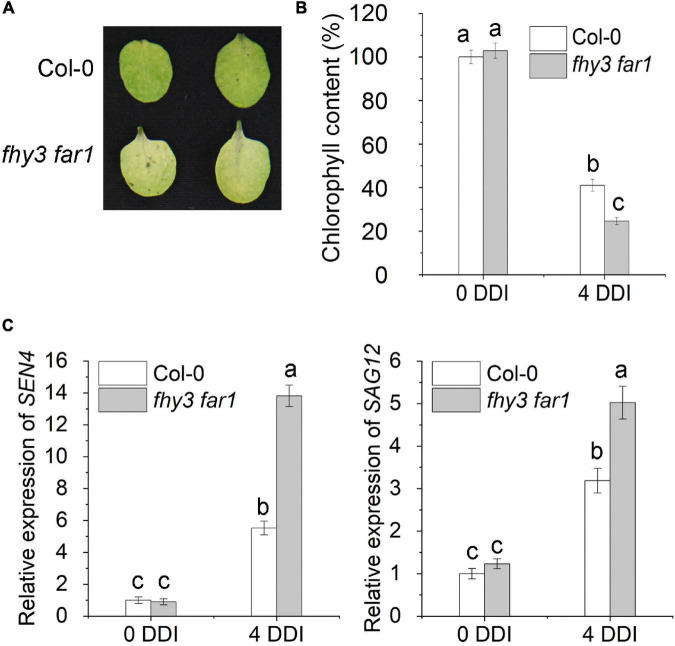
*FHY3/FAR1* negatively regulates leaf senescence upon darkness induction. **(A)** The senescence phenotypes of the detached leaves of 4-week-old Col-0 wild type and *fhy3 far1* double mutant plants incubated under darkness for 4 days (4 DDI). **(B)** The chlorophyll content of the fourth leaves in **(A)**. Error bars represent SD (*n* = 6). Letters indicate significant differences by two-sided LSD test (*p* < 0.05). **(C)** Quantitative RT-PCR analysis of *SEN4* (left) and *SAG12* (right) gene expression in the fourth leaves of Col-0 and *fhy3 far1* without (0 DDI) or with darkness induction for 4 days (4 DDI). Error bars represent SD (*n* = 3). Letters indicate significant differences by two-sided LSD test (*p* < 0.05).

We previously showed that FHY3 and FAR1 proteins physically interact with both EIN3 and PIF5 transcription factors (Liu et al., 2017, 2020), while both EIN3 and PIF5 were reported to up-regulate the expression of *ORE1*, a key NAC transcription factor promoting leaf senescence (Sakuraba et al., 2014; Qiu et al., 2015). Thus, we hypothesized that FHY3 and FAR1 may regulate leaf senescence through modulating the functionality of the *EIN*3-*ORE1* and *PIF5*-*ORE1* transcriptional modules. To test our hypothesis, we first compared the leaf phenotype of the *fhy3 far1* double mutant and wild type plant (Col-0) grown under long-day (16 h light/8 h darkness) conditions. The result showed that the *fhy3 far1* plants indeed showed an obvious early leaf senescence phenotype, with lower chlorophyll contents and higher expression levels of several well-known senescence-induced genes (*SEN4*, *SAG12*, *SAG13* and *SAG29*) in the fourth leaves of 30 and 36 day-old plants compared with those in the same-aged wild type plants (Supplementary Figures 1A–D). Consistent with this, the leaves of *FHY3* overexpressors (*FHY3OE*) senesced later than the wild type plants (Supplementary Figure 2). Since dark treatment is known to induce rapid and synchronous leaf senescence and is adopted to simulate natural senescence (Weaver et al., 1998; Weaver and Amasino, 2001), thus we exposed the third and fourth rosette leaves detached from 4-week-old wild type (Col-0) and *fhy3 far1* mutant plants to darkness. We found that the detached leaves from *fhy3 far1* mutants showed significantly faster senescence than the wild type, with significantly lower chlorophyll content (Figures 1A,B), consistent with their effects on age-dependent senescence. The expression levels of *SEN4* and *SAG12* were also much higher in the *fhy3 far1* mutant than those in wild type plants (Figure 1C). Moreover, with 4-day darkness treatment (4 DDI), 32-day-old *fhy3-11* and *fhy3 far1* plants exhibited early leaf senescence when compared with the same-aged wild type plants, while the *FHY3OE* plants showed late leaf senescence (Supplementary Figure 2). Collectively, these results indicate that FHY3 and FAR1 negatively regulate leaf senescence with or without darkness induction.

It is well known that ethylene promotes leaf senescence, and two closely related transcription factors, EIN3 and EIL1, are essential for ethylene signaling (Chao et al., 1997). It has been shown that EIN3 and EIL1 can directly activate the expression of several senescence-associated genes, such as *ORE1*, *NAP*, and *WRKY75* (Qiu et al., 2015; Guo et al., 2017; Zhang et al., 2017), to promote leaf senescence. We previously showed that FHY3 and FAR1 can directly interact with EIN3 and EIL1 *in vivo* and *in vitro* to coordinately regulate the phosphate starvation response in *Arabidopsis* (Liu et al., 2017). Thus, we examined the available databases of *EIN3* and *FHY3* target genes (Ouyang et al., 2011; Chang et al., 2013; Wang et al., 2016) and identified several senescence associated genes among the co-regulated genes by *FHY3* and *EIN3*, including *WRKY75*, *ORE1*, *NAP*, *SAG20*, and *SAG21*. qRT-PCR analysis verified that the expression patterns of these genes indeed changed in the *fhy3 far1* mutant compared with the wild type (Supplementary Figure 3).

**FIGURE 2:**
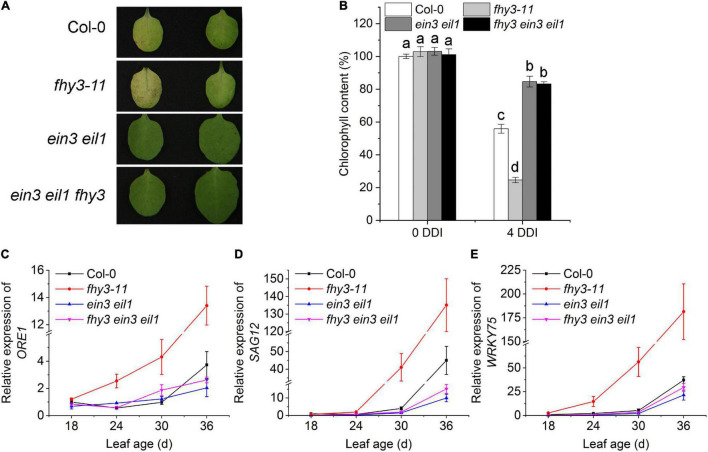
Function of *FHY3/FAR1* in regulating leaf senescence depends on *EIN3/EIL1*. **(A)** The senescence phenotypes of the detached leaves of 4-week-old Col-0, *fhy3-11*, *ein3 eil1*, and *fhy3 ein3 eil1* plants incubated under darkness for 4 days. **(B)** The chlorophyll content of the fourth leaves in **(A)**. Letters indicate significant differences by two-sided LSD test (*p* < 0.05). **(C–E)** Quantitative RT-PCR analysis of the expression of *ORE1* **(C)**, *SAG12* **(D)**, and *WRKY75* **(E)** in the fourth leaves of Col-0, *fhy3-11*, *ein3 eil1*, *fhy3 ein3 eil1* at the indicated leaf age. Error bars represent SD (*n* = 3).

To investigate the genetic interaction between *FHY3/FAR1* and *EIN3/EIL1* in regulating leaf senescence, we constructed the *fhy3 ein3 eil1* triple mutant and compared its leaf senescence phenotype with that of *ein3 eil1*, and *fhy3-11*. We found that after 4 days in darkness, the detached leaves of the *fhy3 ein3 eil1* mutant, but not *fhy3-11*, maintained green just like the *ein3 eil1* parental mutant (Figure 2A). In addition, their distinct leaf yellowing phenotypes of the detached 4-day-old leaves were consistent with the results of a quantitative assay of chlorophyll contents in these mutants (Figure 2B). We also observed a similar senescence pattern for naturally senescence plants with or without darkness treatment (Supplementary Figure 4). Moreover, expression of the senescence associated genes *SAG12*, *ORE1*, and *WRKY75* at the indicated leaf ages were also in consistent with the senescence phenotypes of the dark-treated leaves (Figures 2C–E). Taking together, these results indicate that *FHY3* and *FAR1* act upstream of *EIN3/EIL1* to regulate leaf senescence.

### *ORE1* Acts Downstream of *FHY3* and *FAR1*

**FIGURE 3:**
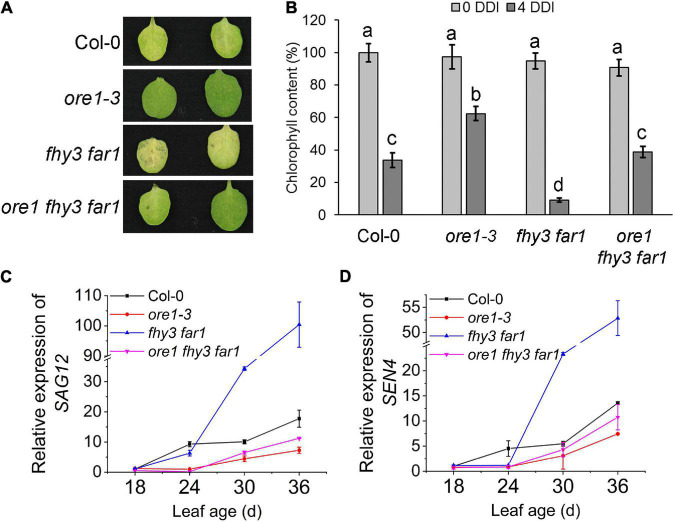
*ORE1* acts downstream of *FHY3* and *FAR1*. **(A)** The senescence phenotypes of the detached leaves of 4-week-old Col-0, *ore1-3*, *fhy3 far1*, and *ore1 fhy3 far1* plants incubated under darkness for 4 days. The *ore1-3* mutant was described in Supplementary Figure 5. **(B)** The chlorophyll content of the fourth leaves in **(A)**. Error bars represent SD (*n* = 6). Letters indicate significant differences by two-sided LSD test (*p* < 0.05). **(C,D)** Quantitative RT-PCR analysis of *SAG12* **(C)** and *SEN4* **(D)** expression in the fourth leaves of Col-0 and *fhy3 far1* at the indicated leaf age. Error bars represent SD (*n* = 3).

Previous studies have shown that *ORE1*, a key regulator of leaf senescence, is a direct target gene of *EIN3* (Qiu et al., 2015). To elucidate the genetic relationship between *ORE1* and *FHY3/FAR1* in controlling leaf senescence, we generated *ore1* single mutant using CRISPR/Cas9 technology (Supplementary Figures 5A,B) and constructed *ore1 fhy3 far1* triple mutant. The fourth detached leaves from two independent *ore1* single mutants, *ore1-3* and *ore1-4*, showed later leaf senescence with darkness treatment than those from wild type plants (Supplementary Figure 5C). The detached leaves of the *fhy3 far1* mutant became yellow after dark treatment for 4 days, whereas the detached leaves of *ore1* and *ore1 fhy3 far1* remained green (Figure 3A and Supplementary Figure 5C). The distinct leaf yellowing phenotypes were consistent with the results of chlorophyll content assay (Figure 3B). Similarly, the 32-day-old plants with or without darkness induction also showed that the *ore1 fhy3 far1* triple mutant exhibited a slower senescence phenotype than the *fhy3 far1* double mutant (Supplementary Figure 5D). Consistently, the expression of *SAG12* and *SEN4* was induced much slower and lower in the *ore1 fhy3 far1* triple mutant compared with the *fhy3 far1* double mutant, similar to the *ore1* single mutant (Figures 3C,D). These observations indicate that *ORE1* acts downstream of *FHY3* and *FAR1* in regulating leaf senescence.

Considering that *WRKY75* is another direct target of *EIN3* (Chang et al., 2013), we also constructed *wrky75 fhy3 far1* triple mutant and observed its senescence phenotype. The leaf senescence phenotype and chlorophyll content showed that the mutation of *WRKY75* partially rescued the early senescence phenotype of *fhy3 far1* (Supplementary Figures 6A,B). Consistent with this, the expression of *SAG12* and *SEN4* was much higher in the 30 and 36-day *wrky75 fhy3 far1* triple mutant compared with those in the same age *wrky75* or wild type, and was close to that in the *fhy3 far1* double mutant (Supplementary Figures 6C,D), suggesting that *WRKY75* may also play a minor role in *FHY3/FAR1-*regulated leaf senescence.

### FHY3 Represses the DNA Binding Activity of EIN3 to the *ORE1* Promoter

**FIGURE 4:**
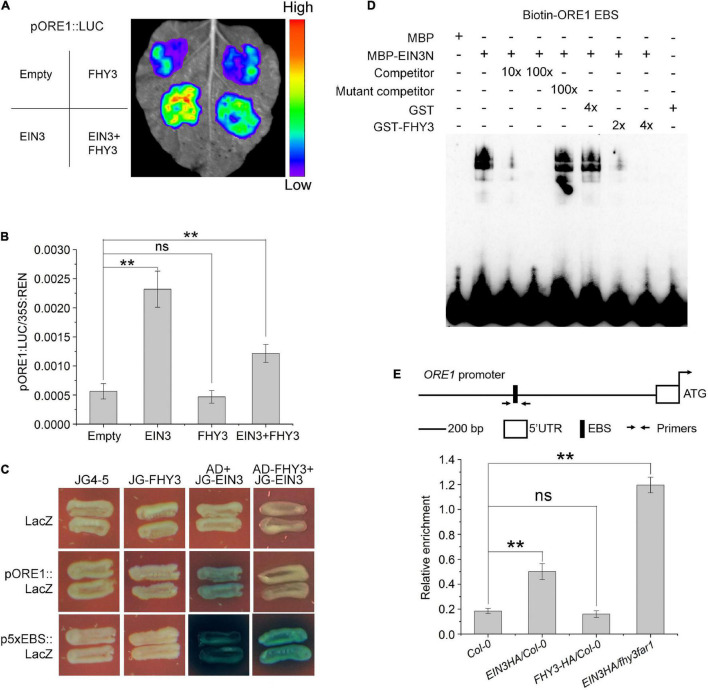
FHY3 repress the DNA binding activity of EIN3. **(A)** Transient expression assay shows that the addition of FHY3 leads to down-regulation of the *ORE1* expression. **(B)** Dual-luciferase assay of LUC expression in **(A)**. The expression of REN was used as an internal control. LUC/REN ration represents the relative activity of the promoters. Values are means ± SD (*n* = 3). Significant difference was identified with Student’s *t*-test. ns, no significance; **p < 0.05. The *pSPYNE-35S* and *pCAMBIA1307* empty vectors were used as controls. **(C)** Yeast one-hybrid assay shows that EIN3 binds to the *ORE1* promoter through the EIN3-binding site (EBS) and FHY3 partially disrupted the interaction between EIN3 and the *ORE1* promoter. 5x EBS, 5 repeats of the EBS in the *ORE1* promoter. **(D)** EMSA results show that FHY3 affects the binding of EIN3 (EIN3N, aa 141–352) to the *ORE1* promoter. Different concentration of GST-FHY3 protein (aa 186–839) was applied (4 μg for lane 7, 8 μg for lane 8). Eight μg of GST was added to lane 6 as a negative control. **(E)** ChIP-qPCR shows the *in vivo* binding of EIN3 to EBS in the *ORE1* promoter. Cross-linked chromatins from EIN3-HA were precipitated with anti-HA antibodies. Eluted DNA was subjected to amplification of the neighboring sequences of EBS by qRT-PCR. Col-0 and *FHY3-HA* plants were used as negative controls. Error bars represent SD (*n* = 3). Significant difference was identified with Student’s *t*-test. ns, no significance; **p < 0.05.

We previously reported that FHY3 and FAR1 can directly interact with the DNA binding domain of EIN3/EIL1 proteins (Liu et al., 2017), we thus speculated whether FHY3 and FAR1 can regulate the function of EIN3/EIL1 during leaf senescence. Results from a dual-luciferase reporter (DLR) system with a 3.5-kb *ORE1* promoter sequence in *Nicotiana benthamiana* leaves (Figures 4A,B) showed that EIN3 alone promoted the transcription of *ORE1*, whereas FHY3 alone seemed have no obvious effect on *ORE1* transcription (Figures 4A,B). However, when *FHY3* was co-expressed with *EIN3* in *N. benthamiana* leaves, the induction of *ORE1* expression by EIN3 was significantly repressed (Figures 4A,B), suggesting that FHY3 represses the transcriptional activation activity of EIN3 on *ORE1*. We next examined whether the physical interaction between FHY3 and EIN3 may affect the DNA binding activity of EIN3. We first used yeast one-hybrid assay to test the effect of FHY3 protein on binding of EIN3 to the EIN3 binding site (EBS, 5′-ATGAACCT-3′, 5x EBS was used here) in the *ORE1* promoter. The results showed that there was no obvious binding between the FHY3 protein and *ORE1* promoter or the 5xEBS fragment (Figure 4C). However, we found that when FHY3 protein was added (construct AD-FHY3), the ability of EIN3 binding to the *ORE1* promoter or the 5xEBS fragment was drastically decreased (Figure 4C). To confirm this, we detected the direct binding activity of EIN3 by electrophoretic mobility shift assay (EMSA). Since the N-terminal fragment of EIN3 (EIN3N, amino acids 141–352) contains the DNA binding domain and has been verified to possess the binding ability (Li et al., 2013), thus we produced the EIN3N proteins and tested its binding activity to the biotin-labeled 60-bp *ORE1* promoter fragment (containing the EBS, designed as Biotin-ORE1 EBS). We found that when FHY3 protein was added, the ability of EIN3 binding to the *ORE1* promoter was drastically decreased (Figure 4D). Since no binding of FHY3 to the *ORE1* promoter was detected (Figure 4C), these results suggest that FHY3 likely regulates *ORE1* expression via the FHY3-EIN3 interaction rather than through direct binding to the *ORE1* promoter. To verify this, we generated transgenic plants expressing HA-tagged *EIN3* (*EIN3-HA*) in the wild type (*EIN3-HA/Col-0*) and *fhy3 far1* double mutant backgrounds (*EIN3-HA/fhy3 far1*). Chromatin immunoprecipitation combined with quantitative PCR (ChIP-qPCR) showed more enrichment of the *ORE1* promoter fragment containing the EBS (Solano et al., 1998) in the *EIN3-HA/fhy3 far1* seedlings, compared to the *EIN3-HA/Col-0* seedlings (Figure 4E). These results indicate that FHY3 represses the DNA binding activity of EIN3 to the *ORE1* promoter.

### FHY3 Represses the DNA Binding Activity of PIF5

**FIGURE 5:**
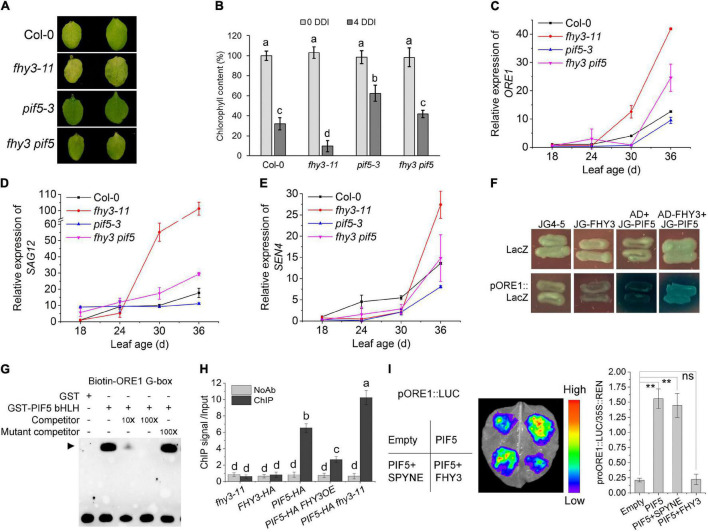
FHY3/FAR1 represses the transcriptional activity of PIF5. **(A)** The senescence phenotypes of the detached leaves of 4-week-old Col-0, *pif5-3*, *fhy3-11*, and *fhy3 pif5* plants incubated under darkness for 4 days. **(B)** The chlorophyll content of the fourth leaves in **(A)**. Error bars represent SD (*n* = 6). Letters indicate significant differences by two-side LSD test (*p* < 0.05). **(C–E)** Quantitative RT-PCR analysis of *ORE1* **(C)**, *SAG12* **(D)**, and *SEN4* **(E)** expression in the fourth leaves of Col-0, *fhy3-11*, *pif5-3*, and *fhy3 pif5* at the indicated leaf age. Error bars represent SD (*n* = 3). **(F)** Yeast one-hybrid assay shows that PIF5 binds to the *ORE1* promoter and that FHY3 interferes with its binding. **(G)** EMSA shows that PIF5 directly binds to the G-box motif in the *ORE1* promoter. **(H)** ChIP-qPCR assay using *p35S:PIF5-HA* transgenic seedlings shows that the enrichment of *ORE1* promoter fragment by PIF5 was inhibited in the presence of FHY3. Leaf tissues from 4-week-old plants were harvested. Values are mean ± SD (*n* = 3). Letters indicate significant differences by two-sided LSD test (*p* < 0.05). **(I)** Transient expression assay using *N. benthamiana* leaves. Tobacco leaves were infiltrated with *A. tumefaciens* transformed with the indicated reporter and effector constructs. The relative LUC activity normalized to REN activity (LUC/REN) are shown. Error bars represent SD (*n* = 3). Significant difference was identified with Student’s *t*-test. ns, no significance; **p < 0.05. The *pSPYNE-3*5S empty vector was used as the control.

Previous studies have shown PIF4 and PIF5 promote leaf senescence by activating the expression of *EIN3*, *ORE1*, *ABI5*, and *EEL* (Sakuraba et al., 2014). Our previous work showed that FHY3 and FAR1 interact with PIF5 both *in vivo* and *in vitro*, and that the bHLH domain of PIF5, which is necessary for DNA binding, is responsible for the interaction with FHY3 (Liu et al., 2020). We thus speculated that FHY3 and FAR1 may also regulate PIF5 function in inducing leaf senescence. To test this possibility, we constructed *fhy3 pif5* double mutant and phenotypic assay showed that, compared with wild type, *pif5-3* leaves displayed a little delayed senescence and the *fhy3 pif5* double mutant exhibited an intermediate phenotype between the *fhy3-11* and *pif5-3* single mutants after dark treatment. Consistent with this, chlorophyll content in the *fhy3 pif5* double mutant was lower than the *pif5-3* single mutant but higher than the *fhy3-11* single mutant (Figures 5A,B). Moreover, the expression patterns of *ORE1*, *SAG12*, and *SEN4* in leaves of these mutants were consistent with their senescence phenotypes (Figures 5C–E). These results suggest that FHY3 acts antagonistically with PIF5 in regulating leaf senescence. In support of this, we found that overexpression of *FHY3* partially repressed the early senescence phenotype of the *PIF5OE* plants (Supplementary Figures 7A,B).

We next tested whether FHY3 can repress PIF5’s DNA binding activity to the *ORE1* promoter. Our yeast one-hybrid assay and EMSA experiments showed that PIF5 could directly bind to the *ORE1* promoter fragment (Figures 5F,G), which is consistent with previous report by Sakuraba et al. (2014), and the addition of FHY3 proteins repressed or interfered with the binding activity of PIF5 (Figure 5F). ChIP-qPCR and DLR assays also verified that PIF5 could directly bind to the *ORE1* promoter and induce expression of *ORE1 in vivo*, while addition of FHY3 repressed PIF5-induced expression of *ORE1* (Figures 5H,I), suggesting that the interaction between FHY3 and PIF5 represses PIF5 binding to the *ORE1* promoter.

### FHY3 Mediates the Formation of a Tri-Protein Complex to Regulate Leaf Senescence

**FIGURE 6:**
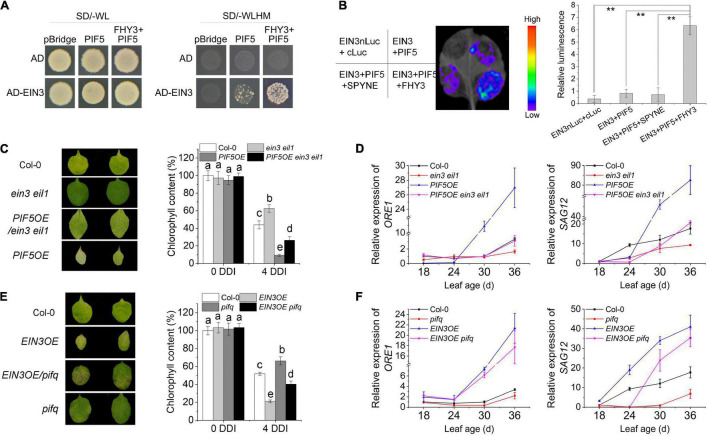
FHY3 and FAR1 mediate the formation of a tri-protein complex to regulate leaf senescence. **(A)** Yeast three-hybrid assay demonstrates that FHY3 bridges a tri-protein complex containing EIN3, FHY3, and PIF5. **(B)** Luciferase complementation imaging (LCI) assay and relative luminescence intensity quantification show that FHY3 enhances the interaction between EIN3 and PIF5 *in vivo*. Error bars represent SD (*n* = 3). Letters indicate significant differences by two-sided LSD test (*p* < 0.05). **(C)** The senescence phenotypes and the chlorophyll content of detached leaves of 4-week-old *ein3 eil1*, *PIF5OE/ein3 eil1*, and *PIF5OE* plants incubated under darkness for 4 days. Letters indicate significant differences by two-sided LSD test (*p* < 0.05). **(D)** Quantitative RT-PCR analysis of *ORE1* and *SAG12* expression in the fourth leaves of *ein3 eil1*, *PIF5OE/ein3 eil1* and *PIF5OE* at the indicated leaf age. Error bars represent SD (*n* = 3). **(E)** The senescence phenotypes and the chlorophyll content of detached leaves of 4-week-old *EIN3OE*, *EIN3OE/pifq* and *pifq* plants incubated under darkness for 4 days. Letters indicate significant differences by two-sided LSD test (*p* < 0.05). **(F)** Quantitative RT-PCR analysis of *ORE1* and *SAG12* expression in the fourth leaves of *EIN3OE*, *EIN3OE/pifq* and *pifq* at the indicated leaf age. Error bars represent SD (*n* = 3).

We previously showed that FHY3 protein directly interacts with both EIN3 and PIF5 (Liu et al., 2017, 2020), thus we wondered whether FHY3, EIN3, and PIF5 can form a tri-protein complex. To test this hypothesis, we conducted yeast three-hybrid experiment and luciferase complementation imaging (LCI) assay in tobacco. Yeast three-hybrid result showed that the interaction between EIN3 and PIF5 could hardly be detected in yeast, but addition of FHY3 obviously increased the interaction between EIN3 and PIF5 (Figure 6A), suggesting that FHY3 may bridge the interaction between EIN3 and PIF5. Similarly, the LCI results showed that the interaction between EIN3 and PIF5 was very weak, but when *FHY3* was co-expressed, the luciferase activity was strongly induced (Figure 6B).

To further investigate the genetic relationship between *PIF5* and *EIN3/EIL1*, we generated the *PIF5OE/ein3 eil1* mutant combination by genetic crosses. Leaf phenotyping, chlorophyll content measurement and downstream senescence-associated genes (*ORE1* and *SAG12*) expression assay all revealed that mutation of *EIN3* and *EIL1* partially rescued the early senescence phenotype of *PIF5OE* (Figures 6C,D). We also generated the *EIN3OE/pifq* mutant. Phenotypic assay showed that its leaves senesced earlier than *pifq* but later than *EIN3OE* (Figure 6E). The expression levels of their downstream targets *ORE1* and *SAG12* in the *EIN3OE/pifq* plants were also intermediate between their parents *EIN3OE* and *pifq* mutants (Figure 6F). These results suggest that *PIF5* likely works in parallel with *EIN3/EIL1*, but downstream of FHY3.

### Regulation of *FHY3* Expression by Developmental Age and Darkness

**FIGURE 7:**
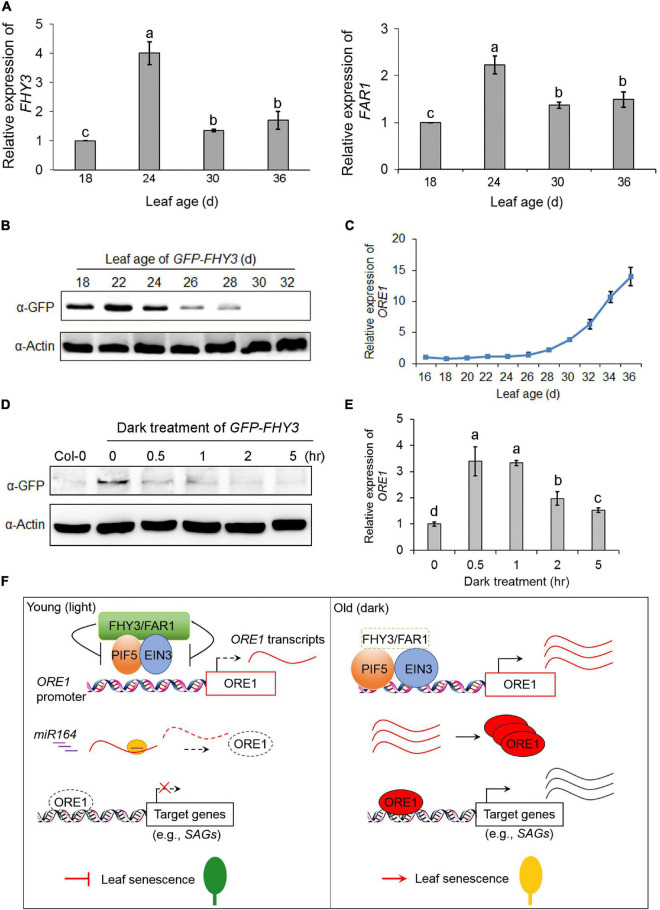
qRT-PCR analysis of FHY3 and FAR1 transcripts and immunoblot analysis of FHY3 protein accumulation. **(A)** qRT-PCR analysis of *FHY3* (left) and *FAR1* (right) expression in the fourth leaves of Col-0 at the indicated leaf age. Error bars represent SD (*n* = 3). Letters indicate significant differences by LSD test (*p* < 0.05). **(B)** Immunoblot assays showing GFP-FHY3 fusion protein levels in the fourth leaves of the *GFP-FHY3* overexpressors at the indicated leaf age. Actin was used as the internal control. **(C)** qRT-PCR analysis of *ORE1* expression in the fourth leaves of the *GFP-FHY3* overexpressors at the indicated leaf age. Error bars represent SD (*n* = 3). **(D)** Western blotting assay showing that FHY3 protein level rapidly declined in seedlings treated with darkness. The *GFP-FHY3* overexpressor seedlings were grown in continuous light for 7 days and then incubated in darkness for the indicated time lengths and then harvested for immunoblot analysis. Anti-GFP antibodies were used to detect FHY3 proteins and actin was adopted as a loading control. **(E)** qRT-PCR analysis of *ORE1* expression in the fourth leaves of the *GFP-FHY3* overexpressors at the indicated points of dark treatment. Error bars represent SD (*n* = 3). Letters indicate significant differences by LSD test (*p* < 0.05). **(F)** A proposed age gating model of *FHY3* and *FAR1* in regulating leaf senescence. In young, green leaves (or under light conditions), FHY3 protein is accumulated above a threshold level, and it represses the DNA binding activity of EIN3 and PIF5 to the *ORE1* promoter, thus repressing *ORE1* expression. In addition, *ORE1* transcript is targeted for degradation by miR164 at a posttranscriptional level. As a result, leaf senescence is inhibited (left). In old leaves (or under darkness), FHY3 and FAR1 protein levels decrease, thus lifting their repression on EIN3 and PIF5, leading to *ORE1* expression. Meanwhile, *ORE1* transcript accumulates due to reduced expression of *MIR164*, allowing translation of ORE1 protein to promote leaf senescence (right).

To investigate how the expression and protein levels of *FHY3* and *FAR1* were regulated during the aging process, we analyzed the expression of *FHY3* and *FAR1* at the four indicated age in wild type (Col-0) leaves. qRT-PCR analysis showed that the expression of *FHY3* and *FAR1* was up-regulated in leaves of about 24-day-old and then down-regulated afterward (Figure 7A). To examine developmental regulation of the FHY3 protein level, we generated transgenic plants expressing GFP-FHY3 fusion protein driven by the 35S promoter (*GFP-FHY3/Col-0*). The functionality of the over-expressed GFP-FHY3 fusion protein was verified by phenotyping under mimicked shade conditions (Supplementary Figure 8). Total protein was extracted from the fourth leaf at the indicated leaf ages and we found that the levels of GFP-FHY3 fusion protein rapidly decreased in leaves older than 24 days (Figure 7B). This result was verified in wild type plants using anti-FHY3 antibodies (Supplementary Figure 9). By contrast, most of the senescence-associated genes (including *ORE1*) were sharply up-regulated in leaves older than 24 days, as previously shown (Figure 7C and Supplementary Figures 1D,E). These results indicate that FHY3 possibly functions at the early stage to repress leaf senescence.

Next, we examined the changes of FHY3 protein levels during dark treatment. The *GFP-FHY3* transgenic seedlings were grown under continuous white light for 7 days and then transferred to darkness for the indicated times. Western blot analysis showed that the FHY3 protein levels decreased quickly upon dark treatment (Figure 7D). By contrast, we observed a rapid up-regulation of *ORE1* gene expression in dark-treated seedlings (Figure 7E).

## Discussion

One effective way to increase crop productivity is to increase planting density. However, high-density planting could trigger shade avoidance syndrome, including exaggerated stem elongation, less branching, early flowering and premature leaf senescence, thus lowering crop productivity (Brouwer et al., 2014). Our previous studies showed that FHY3 and FAR1 play important roles in regulating multiple aspects of shade avoidance response, including hypocotyl elongation, branch number, flowering time, and plant defense (Liu et al., 2019; Xie et al., 2020a,b).

In this study, we collected several lines of evidence to show that FHY3 and FAR1 act as negative regulators of leaf senescence. We showed that *fhy3 far1* mutant senesced earlier (turn yellow earlier, more rapid loss of chlorophyll, and earlier induction of senescence-associated gene expression) than wild type under normal long-day conditions, as well as in dark-treated detached leaves (Figure 1 and Supplementary Figure 1). Genetic epistasis assay showed that *FHY3* and *FAR1* act upstream of *EIN3*, *EIL1*, *PIF5* and *ORE1* (Figures 2–5). We further showed that FHY3 and FAR1 directly interact with EIN3, EIL1, and PIF5 and repress their binding to the *ORE1* promoter (Figures 4, 5; Liu et al., 2020). Moreover, we accumulated evidence suggesting that FHY3, EIN3 and PIF5 can form a tri-protein complex(es) to coordinately regulate leaf senescence (Figure 6). Further, we showed that the protein level of FHY3 is rapidly down-regulated in leaves older than 24 days/or is rapidly down-regulated by dark treatment, concomitant with the observed rapid induction of *ORE1* and other *SAG* genes (Figure 7). Based on these results, we propose a model that FHY3 and FAR1 act as an age gating mechanism to prevent precocious leaf senescence. In young leaves (less than 24 days old) or plants under normal light conditions, FHY3/FAR1 proteins accumulate and they inhibit the DNA binding activities of EIN3 and PIF5 to the *ORE1* promoter through direct physical interaction. As a result, *ORE1* expression is reduced. In addition, the expression of *ORE1* is further negatively regulated by miR164 at a posttranscriptional level in young leaves (Kim et al., 2009). Thus, both transcriptional and posttranscriptional repression of *ORE1* expression may constitute a “double-secure” mechanism to prevent precocious leaf senescence in young leaves. During aging or under dark conditions, FHY3 protein level decreases so that its inhibitory effect on EIN3 and PIF5 is lifted. Meanwhile, the expression of *EIN3* and *PIF5* is up-regulated, while expression of *MIR164* is down-regulated (Kim et al., 2009; Li et al., 2013; Song et al., 2014). These molecular events collectively lead to rapid induction of *ORE1* expression, thus promoting leaf senescence (Figure 7F). Our model is consistent with and provides a mechanistic explanation for the earlier reports that activation of ethylene signaling can only trigger leaf senescence in leaves that have reached a defined age (Buchanan-Wollaston et al., 2005; Jing et al., 2005).

Our previous study demonstrated that both FHY3 and FAR1 directly bind to the promoter of *CIRCADIAN CLOCK ASSOCIATED1* (*CCA1*), a key component of the core oscillator of the circadian clock, and activate its expression, while PIF5 could also directly bind to the *CCA1* promoter but repress its expression (Liu et al., 2020). Furthermore, we showed that PIF5 physically interacts with FHY3 and suppresses its transcriptional activation activity on *CCA1* expression (Liu et al., 2020). On the other hand, it has been demonstrated that CCA1 directly suppresses *ORE1* expression to counteract leaf senescence (Song et al., 2018). In this study we found that *fhy3 pif5* double mutant exhibited an intermediate level of leaf senescence phenotype between the *fhy3-11* and *pif5-3* single mutants and that overexpression of *FHY3* partially repressed the early senescence of the *PIF5OE* plants. Thus there is a probability that the compromised phenotype of the *fhy3 pif5* double mutant is due to the negative effect of PIF5 on transcriptional activity of FHY3 and therefore reduction of *CCA1* expression.

It is also worth noting that earlier studies have found that in the *fhy3 far1* mutant, the levels of both SA and reactive oxygen species (ROS) increased (Wang et al., 2016). Both SA and ROS are known to act as positive regulators of leaf senescence (Buchanan-Wollaston et al., 2005; Rivas-San and Plasencia, 2011). Thus, it is possible that FHY3 and FAR1 may also regulate leaf senescence through the SA and ROS signaling pathways. Interestingly, these studies have shown that FHY3 and FAR1 can directly regulate the expression of *myo-Inositol-1-phosphate synthase1* (*MIPS1*) and *HEMB1* (which encodes a 5-aminolevulinic acid dehydratase in the chlorophyll biosynthetic pathway), and that constitutive expression of *MIPS1* or *HEMB1* can partially or largely rescued the cell death phenotype and oxidative stress in *fhy3 far1* (Ma et al., 2016; Wang et al., 2016). Interestingly, a recent study reported that the transcription factor WRKY75 can promotes SA production by inducing the transcription of *SA INDUCTION-DEFICIENT2* (*SID2*) and suppresses H_2_O_2_ scavenging, partly by repressing the transcription of *CATALASE2* (*CAT2*) (Guo et al., 2017). Similarly, a recent study reported that *FHY3* and *FAR1* regulate leaf senescence by repressing the expression of *WRKY28* and thus suppressing SA biosynthesis (Tian et al., 2020). The detailed molecular mechanism interconnecting *FHY3/FAR1*-mediated transcriptional regulation of *ORE1* with the SA and ROS signaling pathways in coordinating leaf senescence will be an interesting avenue for future research.

Besides ethylene and SA, other phytohormones, including cytokinins, auxins, ABA, and JA are also known to regulate leaf senescence (Lim et al., 2007; Jibran et al., 2013). Particularly worth mentioning, *FHY3* and *FAR1* have been previously shown to regulate multifaceted developmental processes by integrating light signaling with multiple hormone signaling pathways (Wang and Wang, 2015). For example, we previously showed that FHY3 and FAR1 can directly activate the expression of *ABI5* and regulate ABA responses in plants (Tang et al., 2013). We also showed that FHY3 directly interacts with EIN3 and that both of them can directly bind to distinct *cis*-elements on the promoter of *PHOSPHOATE STARVATION RESPONSE1* (*PHR1*) to coordinately regulate light- and ethylene-mediated phosphate starvation response (Liu et al., 2017). We additionally showed that FHY3 can also physically with multiple JASMONATE ZIM-DOMAIN (JAZ) proteins and MYC2, a group of key regulators of JA responses, to coordinately regulate JA-mediated growth and defense responses (Liu et al., 2019). Thus, it is expected that *FHY3* and *FAR1* may regulate leaf senescence through cross talking with these hormone signaling pathways as well. These results on one hand, suggest that FHY3 and FAR1 may indeed act as a signaling hub regulating leaf senescence via integrating various internal and external signals, and on the other hand, call for more detailed research to fully elucidate the detailed molecular mechanisms of FHY3 and FAR1 in regulating leaf senescence.

## Materials and Methods

### Plant Materials and Growth Conditions

The *Arabidopsis thaliana* ecotype Columbia (Col-0) is the parent line for all mutants and transgenic plants used in this study. Transgenic lines in different genetic backgrounds and multiple mutants are constructed by genetic crosses. *fhy3-11* (SALK_002711) and *far1-4* (SALK_031652) were obtained from the ABRC, *ein3 eil1* (Alonso et al., 2003), *pif5-3*, *pifq* (Zhong et al., 2012) were described previously. *FHY3* overexpressors (*FHY3OE*) and *FAR1* overexpressors (*FAR1OE*) have been described in Ma et al. (2017) and Liu et al. (2019), respectively. The *wrky75* mutant has been described in Guo et al. (2017). Double or triple mutants were generated by genetic crosses.

*Arabidopsis* seeds were surface-sterilized and plated on Murashige and Skoog (MS) medium (4.4 g/L MS salts, 1% [w/v] sucrose, pH 5.8, and 8 g/L agar). After stratification at 4°C for 3 days, the seedlings were transferred to soil and grown at 22°C under long-day conditions (16-h light/8-h dark). The white light source was provided by LED (PAR = 100 μmol m^–2^ s^–1^).

### Construction of Plasmids and Generation of Transgenic Plants

For *JG-EIN3*, *JG-PIF5*, and *JG-FHY3* constructs, the individual full-length coding sequences of *EIN3*, *PIF5*, and *FHY3* were ligated to the vector *pB42AD* and designed as *JG-EIN3*, *JG-PIF5*, and *JG-FHY3*, respectively. For *AD-FHY3* construct, the coding sequences of *FHY3* was ligated to the *pEG202* vector (Clontech) and designed as *AD-FHY3*. To create *pORE1:LacZ*, the *ORE1* promoter was amplified from genomic DNA and inserted into *pLacZ2*μ vector (Lin et al., 2007) digested with *Eco*RI and *Xho*I. To construct *p5x EBS:LacZ*, 5 repeats of the *ORE1* promoter fragment containing the EIN3 binding site (5′-aatatactttacaaggttcatgcatgcatacattgttttc-3′) was amplified and inserted into *Sal*I digested *pLacZi2*μ vector (Lin et al., 2007). The five tandem repeats of EBS (5x EBS) were designed in the primer pairs P03 and P04. Two subfragments, 5x EBS-1 (amplified with primer pair P03) and 5xEBS-2 (amplified with primer pair P04), together with *pLacZi2*μ vector (digested with *Eco*RI/*Xho*I) were incubated in 2x Gibson Assembly Master Mix (New England Biolabs) to generate the construct *p5xEBS:LacZ* for yeast one hybrid.

For yeast three-hybrid assay, the coding sequence of *EIN3* was cloned from cDNA into *Eco*RI-digested *pGADT7* vector to generate the *AD-EIN3* construct. *PIF5* coding sequence was amplified from cDNA and cloned into *Eco*RI-digested *pBridge* vector to generate the *pBridge-PIF5* construct. Then *FHY3* coding sequence was amplified from cDNA and inserted into *Bgl*II-digested *pBridge-PIF5* to generate the *pBridge-PIF5-FHY3* construct.

To generate GST-FHY3 (aa 186-TAA), the *FHY3* fragment (aa 186-TAA) was amplified from cDNA and inserted into *Eco*RI digested *pGEX-5x-1.*

Plasmids of the 35S promoter-driven effectors for dual Luc reporter system were described previously (Liu et al., 2017, 2019). To generate *pORE1:Luc*, a 3.5-kb genomic promoter sequence upstream of the coding region of *ORE1* was amplified, and inserted into *Sal*I digested *pGreenII-0800* vector (Hellens et al., 2005).

The oligonucleotide primers for the constructs above are summarized in Supplementary Table 1. The constructs were verified by DNA sequencing analysis.

*FHY3OE* (*35S:FLAG-FHY3-HA*), *PIF5OE* (*35S:PIF5-HA*) were lab stock (Liu et al., 2017; Xie et al., 2017). *FHY3OE PIF5OE* transgenic plants were generated by crossing *FHY3OE* and *PIF5OE*. *PIF5OE/ein3 eil1* transgenic plants were generated by crossing *PIF5OE* and *ein3 eil1*. *GFP-FHY3* (*35S:GFP-FHY3*) transgenic plants were obtained by cloning *FHY3* coding sequences into the *pEGAD* vector and transforming the construct into Col-0 background. Homozygotes were characterized by hygromycin resistance in the T_3_ population. *EIN3-HA* (*35S:EIN3-HA*) transgenic plants were obtained by cloning *EIN3* coding sequences into the *pCAMBIA1307* vector and transforming the construct into Col-0 background. Homozygotes were characterized by hygromycin resistance in the T_3_ population. *EIN3HA/fhy3 far1* transgenic plants were obtained by crossing *EIN3-HA* transgenic plants with *fhy3 far1*, and the homozygotes were characterized by PCR-based genotyping of the F_2_ population.

### Measurement of Chlorophyll Content

Chlorophyll contents were measured in the third and fourth leaves using a SPAD Chlorophyll Meter (SPAD-502 Plus, Konica Minolta). Each leaf was evenly divided into 5–6 spots, and one measurement was taken per spot. The average value of the 5–6 measurements (SPAD Unit) represents a single data point and one biological replicate. Six individual leaves of each genotype are measured, and three biological replicates were performed.

### RNA Extraction, Reverse Transcription, and Real-Time PCR

Total RNA was extracted from the fourth leaf of the indicated leaf ages using Trizol reagent (Invitrogen). Reverse transcription was performed using reverse transcriptase (Tiangen). cDNA was diluted 1:10 and subjected to quantitative PCR using SuperReal PreMix Plus (Tiangen) and a 7500 Real Time PCR System (Applied Biosystems, United States) cycler according to the manufacturer’s manual. The level of *ACT2* transcript was adopted as an internal control. The oligonucleotide primers for Real-time PCR are summarized in Supplementary Table 1.

### Yeast One-Hybrid Assay

To detect the binding of EIN3, FHY3 or PIF5 proteins to the *ORE1* promoter, plasmids of indicated JG-fusion proteins (such as *JG-FHY3*, *JG-EIN3* or *JG-PIF5*) were cotransformed with the indicated *ORE1* promoter reporter plasmids into the yeast strain EGY48. Transformants grown on the SD/-Trp/-Ura medium (Clontech, United States) were transferred to the selection medium containing raffinose, galactose, and 5-bromo-4-chloro-3-indolyl-β-D-galactopyranoside (Amresco, United States) for blue color development. To test the effect of FHY3 on the binding of PIF5 or EIN3 to *ORE1* promoter, *AD-FHY3* and *JG-EIN3* or *JG-PIF5* were cotransformed with the indicated *ORE1* promoter reporter plasmids into the yeast strain EGY48. Transformants grown on the SD/-Ura/-Trp/-His medium (Clontech, United States) were transferred to the selection medium for blue color development.

### Yeast Three-Hybrid Assay

Vectors were cotransfected into the AH109 yeast strain according to the manufacturer’s protocol (Clontech, United States). Yeast were grown on selection plate (SD/-Trp/-Leu) for 3–4 days and then transferred to selection plate (SD/-Trp/-Leu/-Met/-His). Positive interactions were recognized by growth on the SD/-Trp/-Leu/-Met/-His plate.

### Electrophoretic Mobility Shift Assay

Biotin-labeled/unlabeled or mutant *ORE1* and *EIN3* promoter oligonucleotide probes were listed in Supplementary Table 1. *MBP-EIN3N* and *GST-PIF5 bHLH* vectors were constructed as described previously (Liu et al., 2017; Xie et al., 2017). GST, GST-PIF5 bHLH, GST-FHY3 (aa 186-TAA), MBP, and MBP-EIN3N fusion proteins were expressed in the *Escherichia coli* strain BL21. The recombinant proteins were purified using either GST-agarose or amylose resin affinity chromatography. EMSA was performed using a LightShift Chemiluminescent EMSA kit (Pierce, United States) according to the manufacturer’s instructions. Briefly, synthetic DNA oligonucleotide probes labeled with biotin were incubated with the indicated recombinant proteins in the presence or absence of excess amounts of unlabeled competitors for 10 min at room temperature. The DNA-protein complexes were separated on 6% native polyacrylamide gels. To analyze FHY3 protein function, 1, 2, and 4 μg of GST-FHY3 were used.

### Chromatin Immunoprecipitation Combined With Quantitative PCR

Chromatin immunoprecipitation was performed as described previously (Saleh et al., 2008). Briefly, 2 g of leaf tissues from 4-week-old Col-0 (used as a negative control, set to a value of 1), *EIN3-HA*, *EIN3-HA/fhy3 far1* were collected and fixed in 1% formaldehyde for 20 min under a vacuum, followed by neutralization using 0.125 M glycine for additional 5 min. The leaves were then washed for three times with water followed by chromatin isolation. Anti-HA antibodies were added to the sonicated chromatin followed by incubation overnight to precipitate the bound DNA fragments. After salmon sperm-sheared DNA/protein A agarose beads, the bound DNA was eluted and amplified with primers corresponding to sequences in the *ORE1* promoter. Each experiment was performed three times using different pools of seedlings. The oligonucleotide primers for qPCR are summarized in Supplementary Table 1.

### Transient Dual-Luciferase Reporter System

Transient expression in *N. benthamiana* was performed as described previously (Sparkes et al., 2006). *A. tumefaciens* strain GV3101 carrying the reporter plasmid (*pORE1:LacZ* or *p5x EBS:LacZ*) and effector plasmids were cultured in liquid Luria-Bertani medium overnight. The dense cultures were incubated into fresh medium by 1:100 dilution and incubated for 6–8 h. The bacteria were then pelleted at 4,000 rpm for 15 min, and resuspended in an infiltration buffer (5 g/L glucose, 10 mM MgCl_2_, 10 mM MES-KOH, pH 5.7; adding 150 μM acetosyringone right before use) to and OD600 of 0.6. The resuspended agrobacteria containing different constructs were mixed equally and then infiltrated into tobacco leaves using 1 mL syringes without needles. Plants were incubated for 2 or 3 days. Firefly luciferase and Renilla luciferase activities were assayed as described previously (Li et al., 2010).

### Luciferase Complementation Imaging

The firefly LCI assays were performed using *N. benthamiana* leaves. Plasmids for LCI were described previously (Liu et al., 2017, 2019). Both the nLUC- (N-terminal luciferase) and cLUC- (C-terminal luciferase) fusion constructs with or without *pSPYNE-FHY3* (empty vector *pSPYNE* as control) were co-infiltrated into *N. benthamiana* leaves via *A. tumefaciens*-mediated co-infiltration. The infiltrated plants were incubated for 2 or 3 days before examining using the Night SHADE LB 985 Plant Imaging System (Berthold, German).

### Accession Numbers

Sequences of all genes analyzed in this work are available at TAIR under the flowing AGI codes: *ORE1* (AT5G39610), *FHY3* (AT3G22170), *FAR1* (AT4G15090), *PIF5* (AT3G59060), *NAP* (AT1G69490), *WRKY75* (AT5G13080), *SEN4* (AT4G30270), *EIN3* (AT3G20770), *SAG12* (AT5G45890), *SAG13* (AT2G29350), *SAG21* (AT4G02380), *SAG20* (AT3G10985), *SAG29* (AT5G13170), *UBQ10* (AT4G05320), *ACT2* (AT3G18780).

## Data Availability Statement

The original contributions presented in the study are included in the article/Supplementary Material, further inquiries can be directed to the corresponding author/s.

## Author Contributions

HYW, YX, and MM designed the research and wrote the manuscript. MM, YL, BW, YX, HBW, and DK performed the experiments and analyzed the data. All authors contributed to the article and approved the submitted version.

## Conflict of Interest

The authors declare that the research was conducted in the absence of any commercial or financial relationships that could be construed as a potential conflict of interest.

## Publisher’s Note

All claims expressed in this article are solely those of the authors and do not necessarily represent those of their affiliated organizations, or those of the publisher, the editors and the reviewers. Any product that may be evaluated in this article, or claim that may be made by its manufacturer, is not guaranteed or endorsed by the publisher.
