# Supplementary material for: Arabidopsis FHY3 and FAR1 Function in Age Gating of Leaf Senescence
Source: Front Plant Sci. 2021 Oct 28;12:770060. doi: 10.3389/fpls.2021.770060 (PMC8584998; doi:10.3389/fpls.2021.770060)
Supplement: Supplementary file 1 [file Data_Sheet_1.pdf]

Supplementary Material

Supplementary Figures

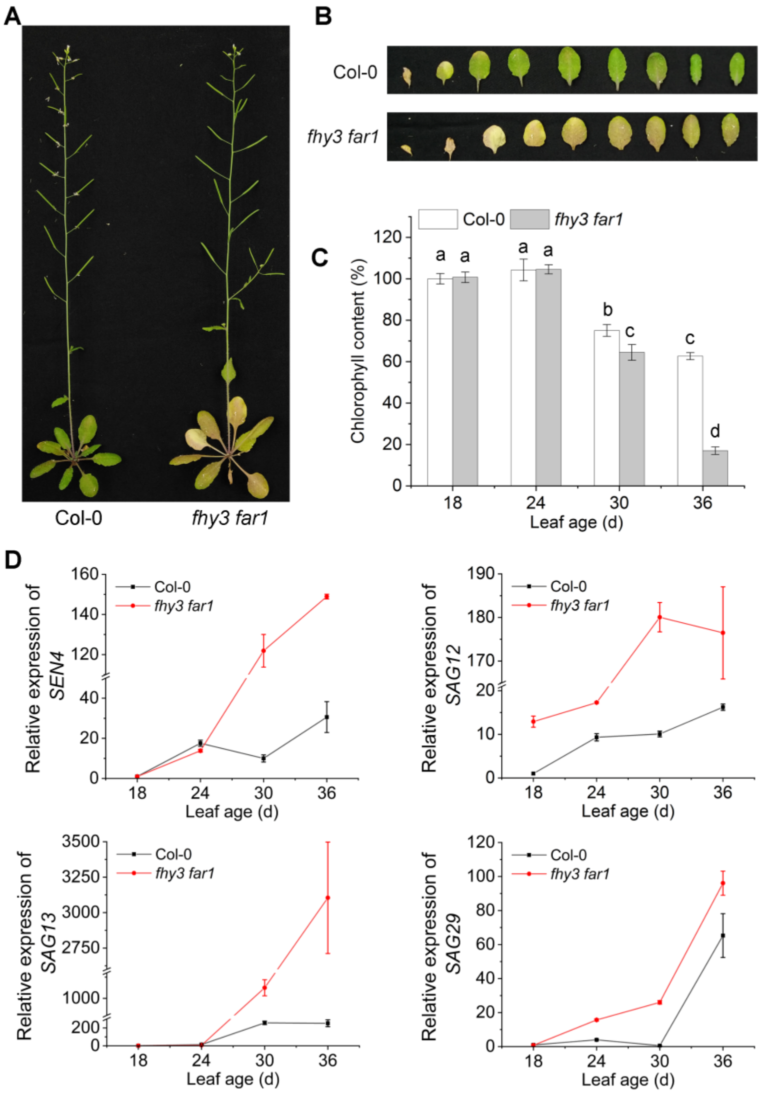

SUPPLEMENTARY FIGURE 1. *FHY3* inhibits leaf senescence under normal conditions. (**A**) The senescence phenotypes of 6-week-old Col-0 and *fhy3 far1* mutant plants grown under normal white light conditions. (**B**) Detached rosette leaves from plants in (**A**). (**C**) The chlorophyll content in the fourth leaves of Col-0 and *fhy3 far1* at the indicated leaf age. Error bars represent SD (n=6). Letters indicate significant differences by LSD test (p<0.05). (**D**) qRT-PCR analysis of the expression of *SEN4*, *SAG12*, *SAG13* and *SAG29* in the fourth leaves of Col-0 and *fhy3 far1* at the indicated leaf age. Error bars represent SD (n=3).

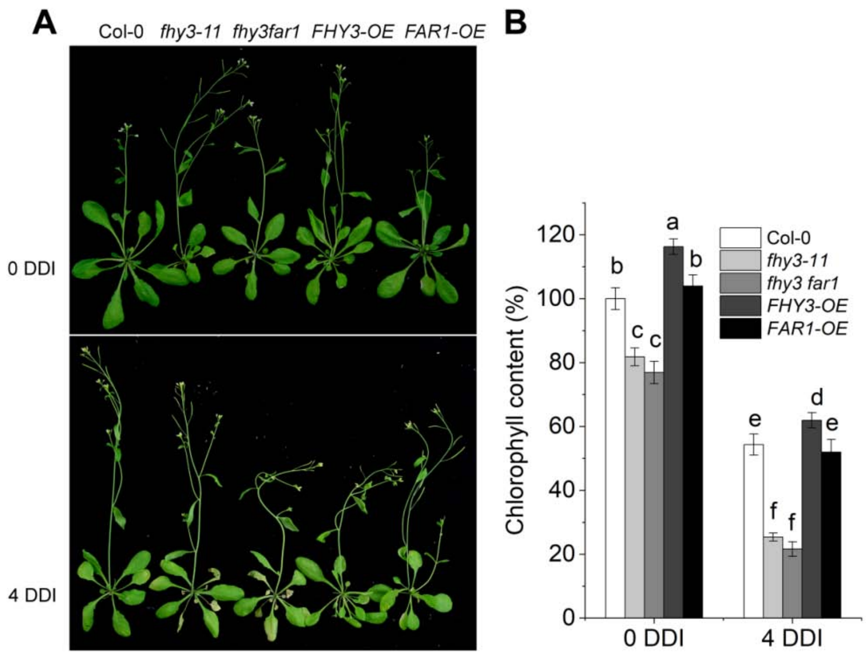

SUPPLEMENTARY FIGURE 2. *FHY3* inhibits leaf senescence upon dark treatment. (**A**) The senescence phenotypes of 32-day-old Col-0, *fhy3-11*, *fhy3 far1*, *FHY3OE* and *FAR1OE* plants without (0 DDI) or with incubation under darkness for 4 days (4 DDI). (**B**) Comparison of the chlorophyll content of the fourth leaves in (A).

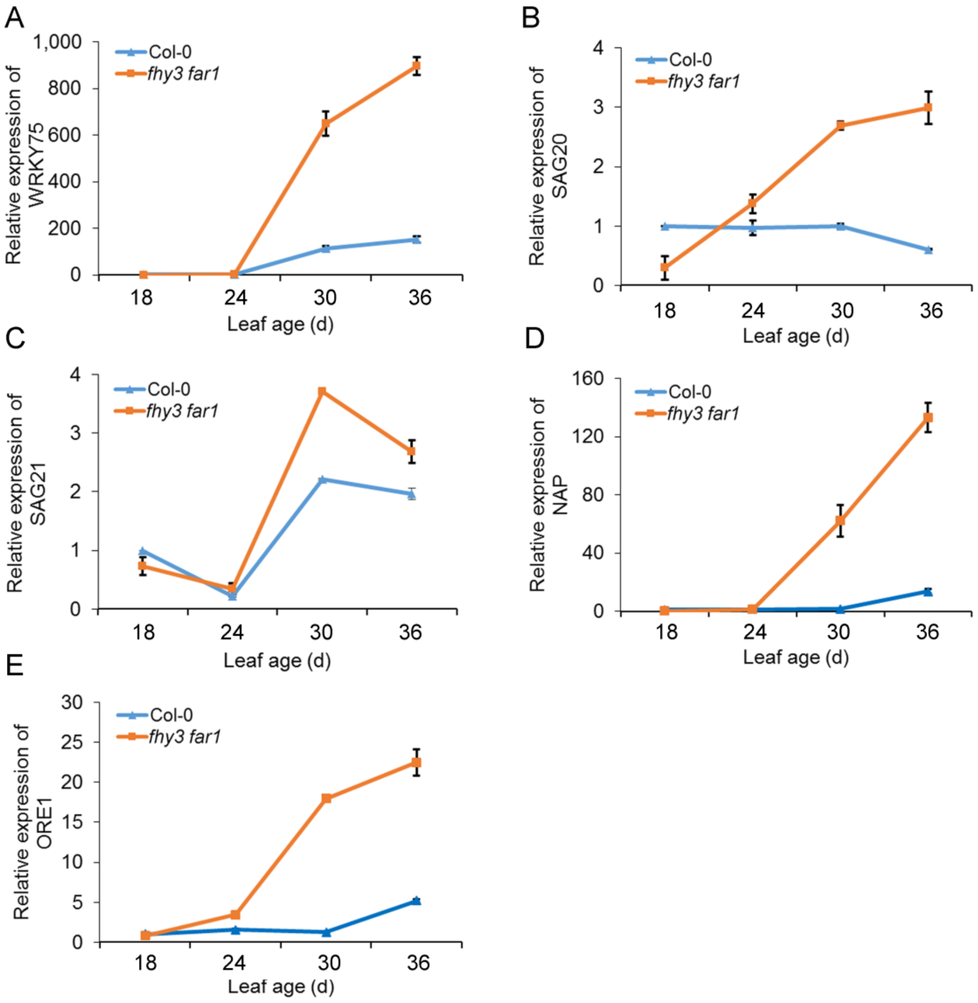

SUPPLEMENTARY FIGURE 3. Representative genes co-regulated by *FHY3/FAR1* and *EIN3/EIL1*. qRT-PCR analysis of *WRKY75* (**A**), *SAG20* (**B**), *SAG21* (**C**), *NAP* (**D**) and *ORE1* (**E**) expression in the fourth leaves of Col-0 leaves at the indicated leaf age. Error bars represent SD (n=3).

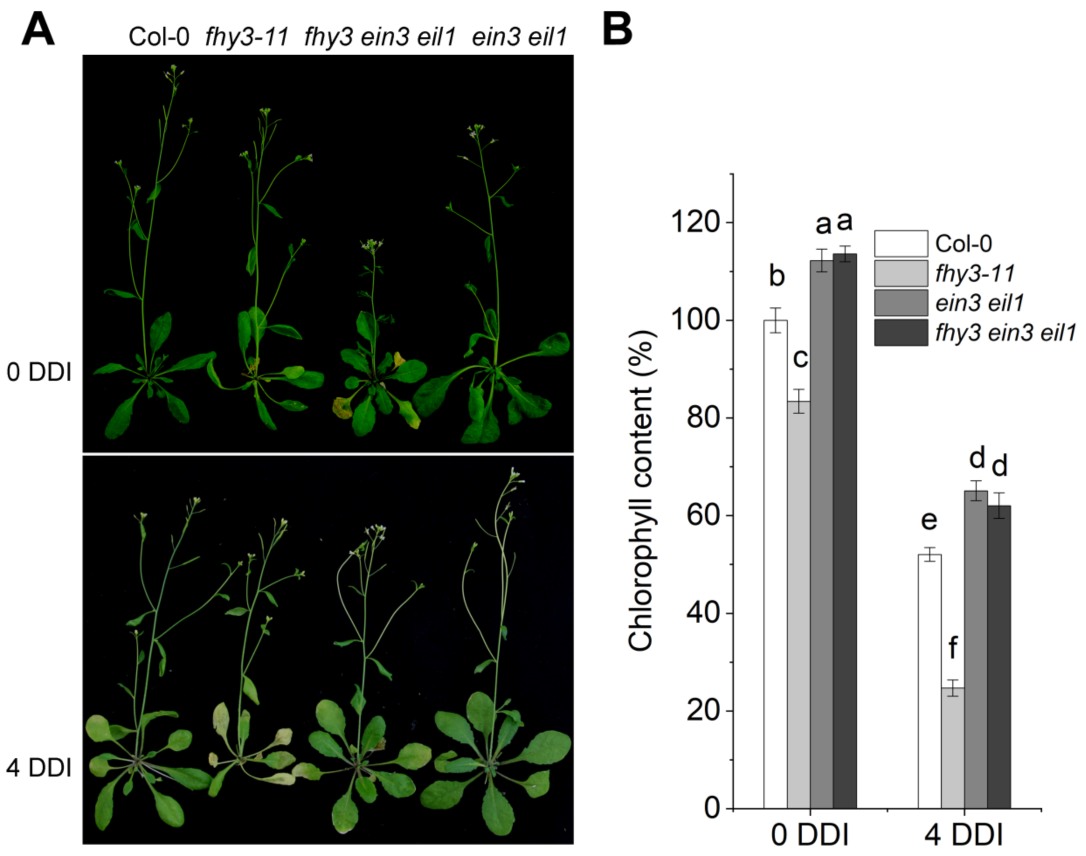

SUPPLEMENTARY FIGURE 4. *EIN3* and *EIL1* act downstream of *FHY3* and *FAR1* in leaf senescence. (**A**) Leaf senescence phenotype of 32-day-old *fhy3-11*, *fhy3 ein3 eil1*, and *ein3 eil1* plants without (0 DDI) or with (4 DDI) incubation under darkness for 4 Days. (B) Comparison of the chlorophyll content of the fourth leaves in (**A**).

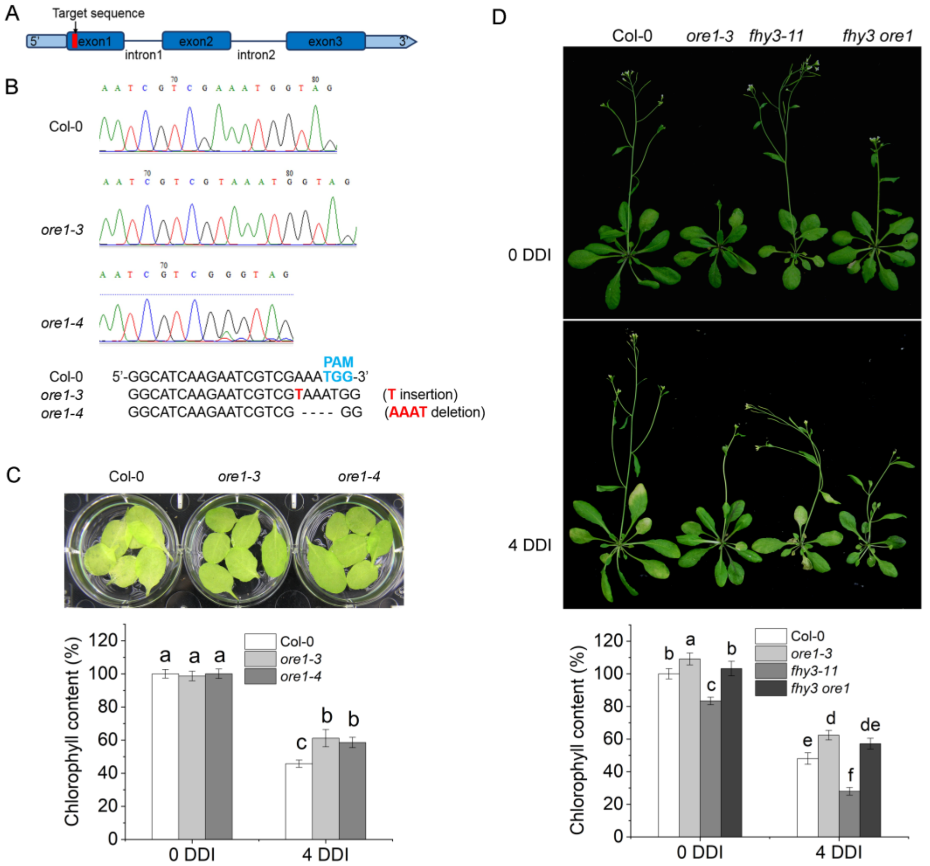

SUPPLEMENTARY FIGURE 5. Generation of two *ore1* mutants using the CRISPR/Cas9 technology. (**A**) Schematic diagram showing the target sequence of the CRISPR-Cas9. The *ORE1* genomic DNA contains three exons and two introns. The PAM (TGG) and the target sequence (GGCATCAAGAATCGTCGAAA) are located in the first exon. (**B**) Sanger sequencing results showing the DNA sequences of the target sequence in Col-0 and two resulting *ore1-crispr* lines (*ore1-3* and *ore1-4*). (**C**) Leaf senescence phenotype and the chlorophyll content of the fourth leaves detached from the *ore1-3* and *ore1-4* plants. Leaves of both lines senesced later than Col-0. *ore1-3* was used to generate the *ore1 fhy3 far1* triple mutant and further experiments shown in Figure 3. (**D**) Leaf senescence phenotype and the chlorophyll content of 32-day-old *ore1-3*, *fhy3-11*, and *fhy3 ore1* adult plants without (0 DDI) or with (4 DDI) incubation under darkness for 4 days.

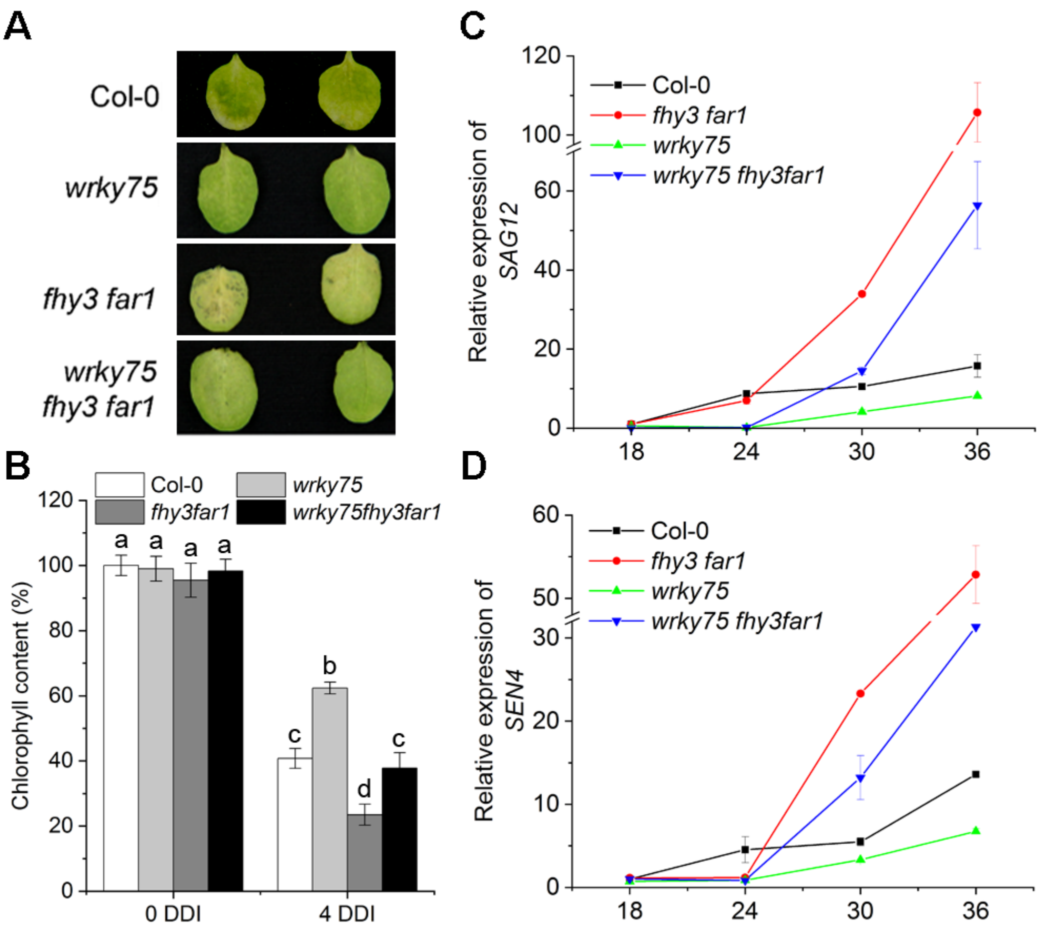

SUPPLEMENTARY FIGURE 6. Mutation of *WRKY75* partially rescues the early leaf senescence phenotype of *fhy3 far1*. (**A**) The senescence phenotypes of detached leaves of 4-week-old Col-0, *wrky75*, *fhy3 far1*, and *wrky75 fhy3 far1* plants incubated under darkness for 4 days. (**B**) The chlorophyll content of the fourth leaves in (A). Error bars represent SD (n=6). Letters indicate significant differences by two-sided LSD test (p<0.05). (**C**) and (**D**) Quantitative RT-PCR analysis of *SAG12* (**C**) and *SEN4* (**D**) expression in the fourth leaves of Col-0, *fhy3 far1*and *wrky75 fhy3 far1* plants at the indicated leaf age. Error bars represent SD (n=3).

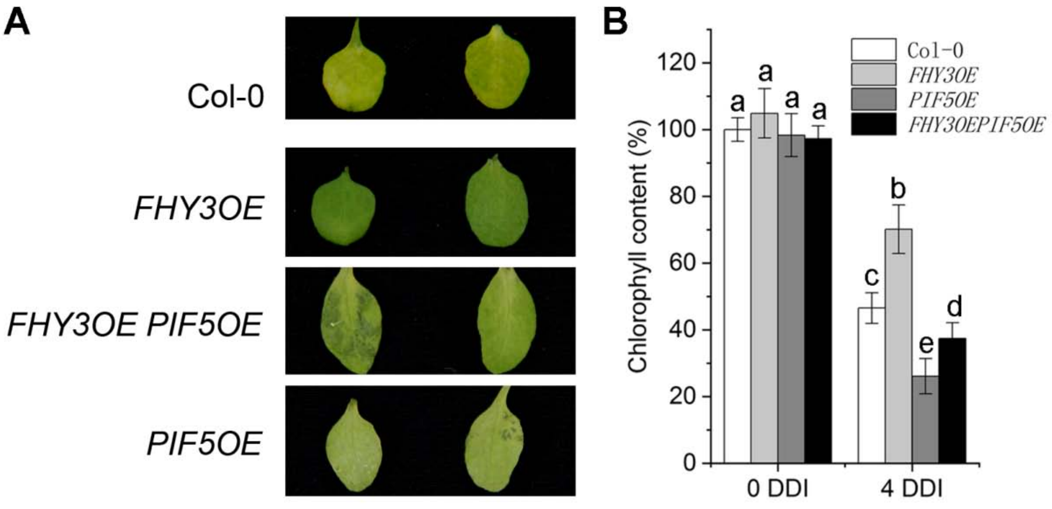

SUPPLEMENTARY FIGURE 7. Over-expression of *FHY3* partially represses the early senescence phenotype of *PIF5OE*. (**A**) The senescence phenotypes of detached leaves of 4-week-old *FHY3OE, FHY3OE PIF5OE* and *PIF5OE* plants incubated in darkness for 4 days. (**B**) The chlorophyll content of the fourth leaves in (**A**). Error bars represent SD (n=6). Letters indicate significant differences by two-side LSD test ($p<0.05$).

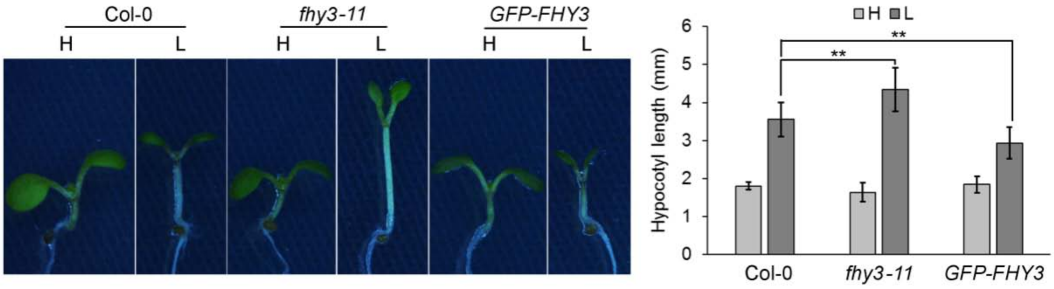

SUPPLEMENTARY FIGURE 8. The *GFP-FHY3* overexpression transgenic seedlings exhibit shorter hypocotyl under simulated shade conditions. Wild-type, *fhy3-11* and *GFP-FHY3* seedlings were grown under white light for 3 days and then either retained in white light (H, High R:FR) or moved to simulated shade (L, Low R:FR)) for 3 days. **, $p<0.01$ by Student's *t* test. Error bars represent SD ($n \geq 15$).

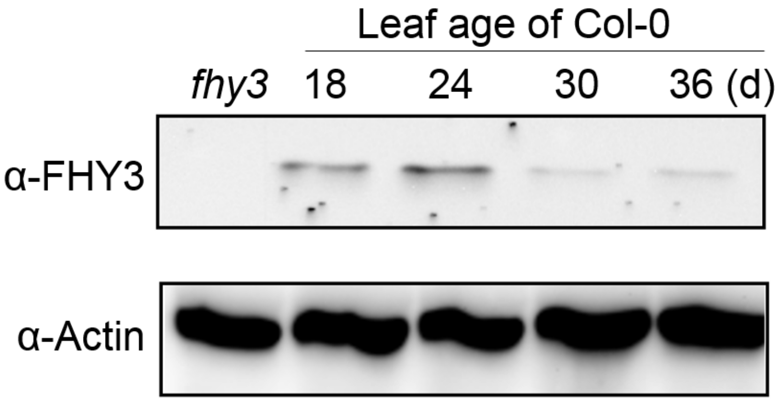

SUPPLEMENTARY FIGURE 9. Immunoblot assay shows that FHY3 protein levels are decreased in the fourth leaves of Col-0 at the indicated leaf age. Anti-FHY3 antibodies were used to detect FHY3 proteins and actin was adopted as the internal control.

Supplementary Table

Supplementary Table 1. Primers Used in This Study

Primer ID	Primer sequence (5'-3')	Purpose
P01	acgatagccatgggcgagctcatgatgtttaatgagatgggaatgtgtggaaac<br>ttttgcggagtacccgggtaccttaagcgtaatctggaacatcgtatgggtagaaccatatggatacatcttgc	Cloning *EIN3-HA* into pCAMBIA1307 vector
P02	tattggatcggaattcaggttatccaattattctcaggtccgttgaattc<br>gagcacatgcctcgagtcctaatagggtttctaaaaatgatcttcttg	Cloning *ORE1* promoter into pLacZ2μ vector
P03	tattggatcggaattcaatatactttacaaggttcatgcatgcatacattgttttcaatatactttac<br>atattgaaaacaatgtatgcatgcatgaaccttgtaaagtatattgaaaacaatgtatgcatgcatgaaccttgtaaagtatattgaaa	Cloning *5x EBS* into pLacZ2μ vector
P04	atacattgttttcaatatactttacaaggttcatgcatgcatacattgttttcaatata<br>gagcacatgcctcgagcaatgtatgcatgcatgaaccttgtaaagtatattgaaaacaatgtatg	
P05	tcgaaggtcgtgggatccccgaattccctgcacaagctgtgagtgaacagaccagaaag<br>ctcgagtcgacccgggaattcttacgagtgtctagacgcgtcctcatgcaactgcgt	Cloning *FHY3* (aa 186-TGA) into pGEX-5x-1 vector
P06	accgggccccccctcgaggtcgactaccatgaaaacaatatgggtatag<br>tatcaagcttatcgataccgtcgacagggtttctaaaaatgatcttcttg	Cloning *ORE1* promoter into pGREEN II 0800 vector
P07	tttaccttccagttttgcaagctagctttcgttgaacaacggaaactcga<br>aatcactacttcgactctagctgtatataaactc	ORE1 knock out by CRIPSR/Cas9 system
P08	cgggctcgagaagcttggatccatggatatagatcttcgactacattcaggtg	Cloning *FHY3*

	agttatctagatccggtggatccttacgagtgtctagacgcgtcctcatgcaac	into pEGADvector
P09	tggccatggaggccagtgaattcatgatgtttaatgagatgggaatgtgtggaaac<br>tcgatgcccacccgggtggaattcttagaaccatatggatacatcttgctgcttc	Cloning *EIN3* into pGADT7 vector
P10	tggcggccgcattagcccgaagatctatggatatagatcttcgactacattcaggtg<br>atgggagatcagcccgaagatctttacgagtgtctagacgcgtcctcatgcaactgcgt	Cloning *FHY3* into pBridge vector
P11	agttgactgtatcgccggaattcatggaacaagtgtttgctgattggaattttgaag<br>aggtcgacggatccccgggaattcttagcctattttacccatatgaagactgtcggt	Cloning *PIF5* into pBridge vector
P12	cagctgcggatgttgttg<br>ccactttctccccattttg	qPCR of *SAG12*
P13	aaggtgacaaagagcaacaattc<br>ctctctaatgggtgtgtcatcg	qPCR of *SEN4*
P14	gccggtttaccttcgttaatgg<br>caaaaccgtctttggactcgtg	qPCR of *ORE1*
P15	ccgtcaagaacaacaagttccc<br>tatgctcgaagttttcggtgga	qPCR of *WRKY75*
P16	gattacatgggacccgtctctc<br>aaacatcgcttgacgatgatgg	qPCR of *NAP*
P17	ggttgaagaaaagaatcgtcgtagg<br>ttagagccaccagtgacaagag	qPCR of *SAG13*
P18	tgatgagtccgttaggtgtgtc<br>acggctcctccactttagtaac	qPCR of *SAG29*
P19	ggtaggagaacatgtgcgaa<br>tatgctccctcacaaagctg	qPCR of *FHY3*
P20	gacattctgcatgcggttag<br>gctgacatcggtctgaagaa	qPCR of *FAR1*
P21	attgttcctccgtgtgggaaag<br>tcctgacgtggctgttacttac	qPCR of *SAG20*

P22	aagggagtggaagaatcaaccc<br>tgcttgttgttcaagagagctg	qPCR of *SAG21*
P23	aaatcacagcacttgcaccaagc<br>ggccttggagatccacatctgc	qPCR of *ACT2* (reference)
P24	tctcgtctctgttatgcttaagaag<br>agaaagaaagagataacaggaacgg	qPCR of *UBQ10* (reference)
P25	atactttacaaggttcatgcatgcatac<br>gtatgcatgcatgaaccttgtaaagtat	*ORE1 EBS* for EMSA
P26	atactttacaagggtaatgcatgcatac<br>gtatgcatgcattacccttgtaaagtat	*ORE1 mEBS* for EMSA
P27	cactgatataccaccacgtgcgcattttggaattgct<br>agcaattccaaaatgcgcacgtggtggtatatcagtg	*ORE1 G box-1* for EMSA
P28	cactgatataccaccccatgcgcattttggaattgct<br>agcaattccaaaatgcgcatggggtggtatatcagtg	*ORE1 mG box-1* for EMSA
P29	gtttcaaagaaaaatcgacacgtgtgtttcgggctcaccaaaactaaga<br>tcttagttttggtgagcccgaaacacacgtgtcgatttttctttgaaac	*ORE1 G box-2* for EMSA
P30	gtttcaaagaaaaatcgacaaatgtgtttcgggctcaccaaaactaaga<br>tcttagttttggtgagcccgaaacacatttgtcgatttttctttgaaac	*ORE1 mG box-1* for EMSA
P31	ctcgtatgaacaaaacacgtgattcgatcatgaaaaga<br>tcttttcatgatcgaatcacgtgttttgttcatacgag	*ORE1 G box-3* for EMSA
P32	ctcgtatgaacaaaacaaatgattcgatcatgaaaaga<br>tcttttcatgatcgaatcatttgttttgttcatacgag	*ORE1 mG box-3* for EMSA
